# Efficacy of Ciprofloxacin in Treating Gram-Negative Infections: Does Obesity Matter?

**DOI:** 10.3390/pharmacy12050147

**Published:** 2024-09-26

**Authors:** Sultan Alotaibi, Nader Damfu, Ahmed Alnefaie, Abdullah Alqurashi, Sami Althagafi, Aown Alotaibi, Musim Alotaibi, Abdullah Alsuwat

**Affiliations:** 1 Department of Pharmaceutical Care Services, King Abdulaziz Medical City, Ministry of the National Guard-Health Affairs, Jeddah 22384, Saudi Arabia; 2 King Abdullah International Medical Research Center, Jeddah 22384, Saudi Arabia; 3 King Saud bin Abdulaziz University for Health Sciences, Jeddah 22384, Saudi Arabia; 4 Infection Prevention and Control Department, King Abdulaziz Medical City, Ministry of the National Guard-Health Affairs, Jeddah 22384, Saudi Arabia; 5 Ashira Al-Eshrafi Health Center, Taif Health Cluster, Taif 21944, Saudi Arabia; 6 Pharmaceutical Care Services, King Faisal Medical Complex, Taif Health Cluster, Taif 21944, Saudi Arabia

**Keywords:** obesity, ciprofloxacin, clinical cure, Gram-negative infections

## Abstract

Background: Obesity is considered a health issue associated with increased morbidity and a risk factor for multiple conditions, such as type 2 diabetes, cardiovascular diseases and infections. It may affect the pharmacokinetics and pharmacodynamics of many drugs, including antimicrobials like ciprofloxacin. Regrettably, data on ciprofloxacin’s efficacy in obese patients remain scarce. This study aims to evaluate the impact of obesity on the efficacy of ciprofloxacin in treating Gram-negative bacterial infections. Methods: A retrospective multicenter cohort study was conducted in two tertiary hospitals in Saudi Arabia. Adult patients (≥18 years) treated with ciprofloxacin for confirmed Gram-negative infection between January 2017 and April 2023 were included. Patients were excluded if they received ciprofloxacin empirically, had inadequate source control within 72 h, or had missing weight and height information at ciprofloxacin initiation. The primary outcome was clinical cure, defined as the resolution of the clinical infection manifestations without additional therapeutic management by the end of treatment. Other secondary and safety outcomes were also assessed. Results: A total of 99 patients were included, divided into obese (*n* = 42) and non-obese (*n* = 57) groups. The obese group had a significantly lower median age (50 years) compared to the non-obese group (64 years) (*p* = 0.002). The obese group had fewer male patients (38.10% vs. 68.42%; *p* = 0.004), higher body weight (90 (81–97) vs. 63 (55–70) days; *p* < 0.001), and lower height (158 (155–165) vs. 165 (158–172) days; *p* = 0.008) compared to non-obese. Urinary tract infection was the most common type, with *Escherichia coli* being the most common isolate. The median hospital length of stay was shorter in the obese group than in the non-obese group (1 vs. 3 days, *p* = 0.007). There were no significant differences in clinical cure rates between obese (85.71%) and non-obese (85.96%) patients (*p* = 1). No significant differences were observed in terms of in-hospital mortality, 30-day mortality, or 60-day infection recurrence rates between the two groups. Microbiological eradication was not achieved in the obese group, whereas a 10.53% eradication rate was observed in the non-obese group (*p* = 0.037). However, the majority of the patients had indeterminate eradication. The incidence of adverse drug reactions (ADRs) was lower in the obese group (4.76%) compared to the non-obese group (17.54%, *p* = 0.066). Conclusions: Treatment with ciprofloxacin in obese patients has similar efficacy and safety outcomes compared to non-obese patients with infections due to Gram-negative pathogens.

## 1. Introduction

Obesity is a serious disease and a significant cause of illness and death. It increases the likelihood of developing type 2 diabetes, recurrent thromboembolism, atrial fibrillation, acute coronary syndrome, and infection [1]. By 2030, more than half of the general population will be obese, and more than 10% will have morbid obesity [2]. Nonetheless, the changed physiology of obesity may have substantial implications on the pharmacokinetics and pharmacodynamics (PK/PD) of antimicrobials, sufficient to require adjustments to dosage regimens. Specifically, increases in volume distribution (Vd) are often reported in obese individuals as a consequence of increased adipose and lean muscle mass [3].

Ciprofloxacin, one of the fluoroquinolones antibiotics, is considered lipophilic in nature, plays a vital role due to its permeability through cell membranes and impacts the drug activity in the human body [4]. The data on ciprofloxacin pharmacokinetics in obesity are contradictory. When healthy volunteers (mean BMI 19.8 kg/m^2^) and obese volunteers (mean BMI 41.0 kg/m^2^) were given the same IV dose, Hollenstein et al. found no difference in Vd or CL [5]. However, one study showed increased Vd and CL (mean obese BMI 36.4 ± 3.9 kg/m^2^; mean control BMI 23.3 ± 2.4 kg/m^2^) [6]. A recent pharmacokinetics study looked at 20 morbidly obese patients (median BMI 45.8 kg/m^2^) and 8 non-obese patients (median BMI 21.42 kg/m^2^), who received one to two doses of ciprofloxacin by IV and oral routes, and they found that body weight did not affect ciprofloxacin bioavailability, CL, or Vd [7]. Dosing in continuous renal replacement therapies (CRRT) was tested in 11 critically ill septic patients using Monte Carlo simulations. They studied different ciprofloxacin dosing regimens on multiple patients’ weight groups (50 kg, 90 kg and 140 kg). The results showed that the maximum dose of 400 mg every 8 h is most likely to meet pharmacodynamic goals in patients with higher body weight and suspected hard-to-treat pathogens [8].

Because drug approval regulatory authorities do not mandate specific trials to assess therapeutic products in obese patients, there are a lack of data regarding the clinical efficacy and safety of ciprofloxacin in this population. As a result, clinicians are increasingly faced with the dilemma of using ciprofloxacin in obese patients [9].

Given the information above, it seems that ciprofloxacin doses for obese patients may be inadequate. The purpose of this study was to evaluate the impact of obesity on the efficacy and safety of ciprofloxacin for treating Gram-negative bacterial infections.

## 2. Materials and Methods

### 2.1. Study Design and Setting

This retrospective multicenter cohort study was conducted among two tertiary hospitals in Saudi Arabia, specifically at the National Guard Health Affairs medical cities in Jeddah and Riyadh. The study aimed to compare the impact of obesity on ciprofloxacin efficacy in obese versus non-obese patients infected by Gram-negative bacterial infections. The study period was from January 2017 to April 2023.

### 2.2. Inclusion and Exclusion Criteria

The study included all adult patients (≥18 years) who received ciprofloxacin as an inpatient for a minimum of 72 h and had isolated cultures confirming Gram-negative infections. Patients were excluded if they received ciprofloxacin empirically, lacked source control within 72 h, received ciprofloxacin as prophylaxis or had missing weight and height documentation within 72 h before or after ciprofloxacin initiation.

### 2.3. Sample Size and Data Collection

**Figure 1:**
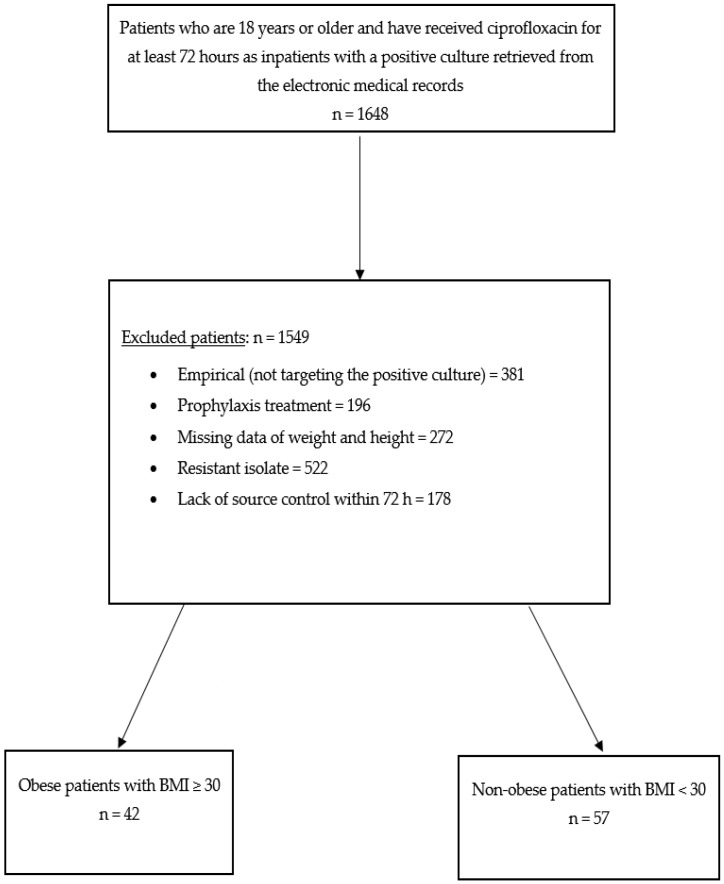
Patient enrollment and screening for eligibility.

The initial cohort consisted of 1648 patients who were admitted to the hospital and received ciprofloxacin. After applying the exclusion criteria, 1549 patients were excluded, leaving a study cohort of 99 patients, divided into obese (*n* = 42) and non-obese (*n* = 57) groups (Figure 1). Data were extracted from electronic health records, encompassing demographics, baseline characteristics, comorbidities, severity of illness, infectious diseases consultation, relevant laboratory values, type of infection, concurrent and previous antibiotic use, microbiological and susceptibility data, and other concurrent infections. Clinical cure was assessed through a review of progress notes documented by treating clinicians, vital signs, laboratory data, and radiographic findings. The clinical cure was further evaluated by a specialized clinical pharmacist in infectious diseases. Safety outcomes were assessed by using the RIFLE criteria for renal function and the documentation of ADRs in the primary physician notes. 

### 2.4. Key Definitions Used in the Study

Clinical cure was defined as the resolution of clinical manifestations associated with the infection, confirmed by the absence of the need for additional therapeutic management for Gram-negative infections due to failure or adverse effects by the end of therapy. Time to study drug was defined as the number of hours from the collection of the first index cultures to the initiation of ciprofloxacin. In-hospital mortality was defined as death occurring for any reason during the hospital admission. Thirty-day mortality was defined as death occurring within 30 days of the initiation of ciprofloxacin, and considered infection-related if patients had ongoing clear clinical or biochemical signs of infection at the time of their death. Microbiological eradication was defined as the negative result of the repeated culture from the site of infection. The polymicrobial infection was defined as the presence of at least one additional pathogenic microorganism during the same episode of the index infection. Sixty-day infection recurrence was defined as the presence of clinical signs and symptoms of infection with repeated isolation of the same pathogen within 60 days of the primary infection episode. Obesity was defined as a body mass index (BMI) of ≥30.

### 2.5. Microbiologic Testing

In this retrospective study, susceptibility results of bacterial isolates were obtained from samples previously collected during routine clinical care at our institutions. Only Gram-negative bacterial isolates confirmed to be susceptible to ciprofloxacin, using the VITEK^®^ MS MALDI-TOF and VITEK^®^ 2 systems (bioMérieux, Marcy-l’Étoile, France), were included.

### 2.6. Statistical Analysis

Categorical variables were expressed as numbers and percentages and compared using the chi-square test or Fisher’s exact test. Continuous variables were presented as median and interquartile range (IQR) and compared using the Mann–Whitney U test. A multivariate logistic regression model was used to identify the predictors of clinical cure events. Covariates were identified through clinical considerations and relevant baseline clinical characteristics associated with a *p* value < 0.1 in the univariate analysis. To prevent multicollinearity, the variance inflation factor (VIF) was calculated for every variable included and considered acceptable if VIF values were <5. Model discrimination was assessed with the area under the receiver operating characteristic curve (AUC), and model calibration was assessed with the Hosmer–Lemeshow test. Statistical results were considered significant using a two-tailed *p* value of less than 0.05. All statistical analyses were performed using SPSS software version 26.0 (IBM, Chicago, IL, USA).

## 3. Results

The study included 99 patients, of whom 42 were obese (BMI ≥ 30) and 57 were non-obese (BMI < 30). The median age in the obese group was significantly lower at 50 years compared to the non-obese group at 64 years (*p* = 0.002). The distribution by gender, height, and actual weight was significantly different between groups, with obese patients characterized by fewer male patients (38.10% vs. 68.42%; *p* = 0.004), a higher median body weight of 90 (81–97) vs. 63 (55–70) days (*p* < 0.001), and a lower median height of 158 (155–165) vs. 165 (158–172) days (*p* = 0.008) than those who are not obese. Baseline serum creatinine levels and the prevalence of most comorbid conditions did not differ significantly, although the Charlson comorbidity index was higher in non-obese patients (median 4 vs. 2, *p* = 0.001). Most of the infections were due to urinary tract infection (UTI), which accounted for 69.05% and 57.89% of the cases in obese and non-obese patients, respectively. Other sources of infection included osteomyelitis, intra-abdominal infections, and acute bacterial skin and skin structure infections. The isolates most commonly found included *Escherichia coli*, *Klebsiella pneumoniae*, and *Pseudomonas aeruginosa*. The type distribution of infections and causative pathogens revealed no significant difference between the obese and non-obese groups. More information is detailed in Table 1.

Clinical cure at end of treatment was also similar; 85.71% of the obese were clinically cured, and 85.96% of the non-obese were clinically cured (*p* = 1). Microbiological eradication was not achieved in any of the obese patients, compared to 10.53% eradication in non-obese patients (*p* = 0.037). However, most of the patients in both groups were indeterminate about microbiological eradication. When comparing the two groups, no statistically significant difference was observed in overall hospital mortality (7.02% vs. 2.38%; *p* = 0.392). Similarly, 30-day all-cause mortality and 30-day and 60-day recurrence rates did not differ significantly between the two groups. The length of stay of hospitalization from the initiation of ciprofloxacin to discharge was significantly shorter in obese patients compared to non-obese patients (median = 1 vs. 3 days; *p* = 0.007). ADRs were less reported in the current study, with a prevalence of 4.76% in obese patients and 17.54% in non-obese patients (*p* = 0.066). The incidence of acute kidney injury, *Clostridioides difficile* infection, hepatotoxicity, nausea and vomiting, seizures, and thrombocytopenia did not significantly differ between the two groups. None of the obese group discontinued ciprofloxacin due to ADRs, while 1.75% of non-obese patients discontinued treatment due to adverse events (Table 2). The relationship between different clinical factors and incidence of clinical cure was evaluated using multivariate logistic regression analysis. The concurrent antibiotic use, lymphoma, overall duration of hospital stay, time to administration of study drug and antibiotic used prior to ciprofloxacin with activity against the isolated organism met the enrollment criteria for the multivariate logistic regression model. However, the multivariate analysis showed that none of these factors remained significantly associated with clinical cure events when adjusted for other variables (Table 3).

## 4. Discussion

According to our knowledge, this is the first study to specifically compare the clinical efficacy of ciprofloxacin between obese and non-obese patients. The findings of this study showed that ciprofloxacin is equally effective at achieving clinical cure in obese and non-obese groups. Moreover, there were no significant differences regarding mortality rates or recurrence of infection. While contrasting these results, Longo et al. reported that obesity was a significant predictor of antibiotic treatment failure, potentially leading to higher recurrence rates [10]. Moreover, Barber and his colleagues reported higher rates of treatment failures in obese patients treated with ceftriaxone therapy compared to non-obese patients [11]. This difference could be due to variations in study design, population characteristics, or infection management protocols, highlighting the need for further prospective studies to clarify these findings. 

Van Rhee et al. also reported that the pharmacokinetics of ciprofloxacin were not significantly influenced in obese patients; its therapeutic effectiveness could be successfully preserved with appropriate dose adjustments. This suggests that ciprofloxacin can be effectively used across different BMI categories with careful monitoring and dosage adjustments without compromising clinical outcomes [7]. However, another study conducted by Allard et al. found that ciprofloxacin clearance is significantly higher in obese patients, necessitating higher doses to achieve therapeutic levels [6]. In this current study, we could not assess the impact of different doses on the clinical cure as a larger sample size is needed. However, the median doses of ciprofloxacin were not significantly different between the two groups.

A unique finding in the current study is the shorter hospital length of stay observed in obese patients (median 1 day) compared to non-obese patients (median 3 days). This finding diverges from the general expectation that obese patients might require longer hospitalizations due to complications related to both obesity and infection management [12]. This shorter length of stay could be attributed to another hypothesis, which is that obese patients in the current study started on ciprofloxacin when they were stable and ready for discharge, highlighting an area for further investigation. Moreover, UTI was the most common infection in both groups, as it is mostly caused by Gram-negative organisms and ciprofloxacin is the preferred oral agent to discharge the patients on it.

In terms of safety, the present study reported a lower occurrence of ADRs among obese patients (4.76%), compared to 17.54% in non-obese patients. The current study is in disagreement with Modesto et al., who reported that one-third of their included obese patients experienced ADRs, which highlights the importance of careful medication management in obese patients due to their susceptibility to ADRs driven by complex therapeutic regimens and multiple health conditions [13]. It is worth mentioning that this variation may be due to the criteria used for documenting ADRs in our study or a difference in individual responses to ciprofloxacin.

Our study found no microbiological eradication in obese patients compared to a 10.53% eradication rate in non-obese patients. The lower eradication rates observed might be attributed to several factors, including difficulty ensuring sufficient ciprofloxacin tissue concentrations in the obese. Studies such as those by Hollenstein et al. and Alobaid et al. have highlighted the difficulties associated with achieving adequate drug levels within adipose tissues [3,4,5]. Regrettably, no available studies that examined the influence of obesity on bacterial eradication were identified in the literature. Nevertheless, the current study finding should be interpreted with caution as the majority of the patients had an indeterminate eradication outcome.

Our study has several limitations. The retrospective design may introduce selection bias and potential confounding factors not accounted for in the analysis. The relatively small sample size of obese patients limits the generalizability of the findings. Additionally, the study did not account for variations in ciprofloxacin dosing regimens or adherence to treatment protocols, which could influence the outcomes. Different sources of infection may create heterogeneity in the results of the study. Data on specific MICs were not documented; however, we only included cases with identified and susceptible pathogens to ciprofloxacin. We rely on physician notes regarding the incidence of ADR, which may underestimate the incidence of ADR between two groups. Finally, most of the patients in both groups have indeterminate microbiological eradication, which might explain the lower documented eradication rates observed in the current study.

## 5. Conclusions

This study showed that ciprofloxacin efficacy and safety in treatment of Gram-negative infections were not statistically different between obese and non-obese patient study groups. Only length of hospital stay outcome was lower in obese group.

## Figures and Tables

**Table 1:** Patient demographic and clinical characteristics.

Characteristic	Non-Obese (n = 57)	Obese (n = 42)	*p*-Value
Age (years)	64 (52–74)	50 (39–63)	0.002
Male n (%)	39 (68.42%)	16 (38.10%)	0.004
Height (cm)	165 (158–172)	158 (155–165)	0.008
Actual Body Weight (kg)	63 (55–70)	90 (81–97)	<0.001
Baseline Scr (µmol/L)	72 (58–103)	68 (58–79)	0.129
Comorbidity, n (%)			
Cerebrovascular disease	5 (8.77)	1 (2.38)	0.237
Chronic heart failure	7 (12.28)	1 (2.38)	0.133
Chronic obstructive pulmonary disease	2 (3.51)	1 (2.38)	1
Dementia	4 (7.02%)	1 (2.38)	0.392
Diabetes mellitus	28 (49.12)	20 (47.62)	0.882
Hemiplegia or paraplegia	1 (1.75)	0 (0%)	1
Hypertension	27 (47.37)	11 (26.19)	0.22
Liver disease	6 (10.53)	2 (4.76)	0.461
Lymphoma	2 (3.51)	2 (4.76%)	1
Leukemia	3 (5.26%)	0 (0%)	1
Moderate to severe chronic renal failure	8 (14.04)	3 (7.14)	0.346
Neurological disease	6 (85.71)	1 (2.38)	0.233
Peripheral vascular disease	2 (3.51)	1 (2.38)	1
Solid Tumor	8 (14.04)	6 (14.29%)	0.971
Charlson comorbidity index	4 (2–6)	2 (0.25–4)	0.001
Immunosuppression, n (%)	10 (17.54%)	7 (16.67%)	0.909
Active chemotherapy	8 (80%)	5 (71.43%)	
Neutropenia (ANC < 500)	1 (10%)	1 (14.29%)	
Solid Organ Bone Marrow Transplant	1 (10%)	1 (14.29%)	
Type of infection, n (%)			
Acute Bacterial Skin and Skin Structure Infections	3 (5.26%)	2 (4.76%)	1
Intra-abdominal Infections	5 (8.77%)	4 (9.52%)	1
Osteomyelitis	6 (10.53%)	3 (7.14%)	0.729
Urinary Tract Infections	33 (57.89%)	29 (69.05%)	0.257
Other	14 (24.56%)	5 (11.90%)	0.114
Causative Pathogen, n (%)			
* Enterobacter cloacae*	6 (10.53%)	3 (7.14%)	0.729
* Escherichia coli*	24 (42.11%)	23 (54.76%)	0.212
* Klebsiella pneumoniae*	11 (19.30%)	9 (21.43%)	0.794
* Pseudomonas aeruginosa*	12 (21.05%)	6 (14.29%)	0.388
Other pathogens (combined)	4 (7.02%)	3 (7.14%)	1
Concurrent Polymicrobial Infections, n (%)	5 (8.77%)	1 (2.38%)	0.237
* Enterococcus faecium*	1 (20%)	0 (0%)	
* Klebsiella pneumoniae*	2 (40%)	0 (0%)	
Methicillin-resistant *Staphylococcus aureus*	2 (40%)	1 (100%)	
Presence of Bacteremia, n (%)	6 (10.53%)	3 (7.14%)	0.729
Antibiotic Used Before Ciprofloxacin with activity against the isolated organism in the same course, n (%)	25 (43.86%)	11 (26.19%)	0.071
Time to Study Drug (hours)	36 (24–72)	30 (24–47)	0.249
Total Daily Dose in mg (e.g., 1000)	1000 (800–1000)	1000 (1000–1000)	0.198
Concurrent antibiotic for more than 48 h with in vitro activity against the causative pathogen, n (%)	5 (8.77%)	1 (2.38%)	0.237
Duration of Treatment (days)	7 (7–10)	7 (7–10)	0.949
Overall Duration of Hospitalization (days)	9 (4–16)	6 (2–12)	0.121
ICU Admission at Infection Onset, n (%)	1 (1.75%)	2 (4.76%)	0.573
Septic shock, n (%)	1 (1.75%)	1 (2.38%)	1
Mechanical Ventilation, n (%)	2 (3.51%)	1 (2.38%)	1
Infectious Disease Consultation, n (%)	10 (17.54%)	5 (11.90%)	0.439

**Table 2:** Study outcomes.

Outcomes	Non-Obese (n = 57)	Obese (n = 42)	*p*-Value
Clinical cure by End of Treatment	49 (85.96%)	36 (85.71%)	1
Microbiological outcome			
Persistent Infection	5 (8.77%)	3 (7.14%)	1
Eradication	6 (10.53%)	0 (0%)	0.037
Indeterminate	46 (80.7%)	39 (92.86%)	0.086
Overall In-Hospital Mortality	4 (7.02%)	1 (2.38%)	0.392
30-day All-Cause Mortality	2 (3.51%)	1 (2.38%)	1
Infection-Related Mortality	1 (1.75%)	1 (2.38%)	1
60-day Recurrence	2 (3.51%)	3 (7.14%)	0.447
Hospital LOS from Start of Ciprofloxacin to Discharge (days)	3 (1–9)	1 (1–2)	0.007
Adverse Drug Reaction	10 (17.54%)	2 (4.76%)	0.066
Acute Kidney Injury	3 (5.26%)	1 (2.38%)	
*Clostridioides difficile* Infection	2 (3.51%)	0 (0%)	
Hepatotoxicity	2 (3.51%)	0 (0%)	
Nausea and vomiting	0 (0%)	1 (2.38%)	
Seizure	2 (3.51%)	0 (0%)	
Thrombocytopenia	1 (1.75%)	0 (0%)	
Discontinuation Due to Adverse Event	1 (1.75%)	0 (0%)	1

**Table 3:** Multivariate logistic regression analysis with clinical cure events as dependent variable *^a^*.

Variable	Univariate		Multivariate	
	OR (95% CI)	*p*-Value	OR (95% CI)	*p*-Value
Concurrent antibiotic for more than 48 h	0.13 ([0.02–0.74])	0.022	0.34 ([0.05–2.41])	0.279
Lymphoma	0.38 ([0.13–1.06])	0.064	0.10 ([0.01–1.12])	0.062
Overall duration of hospitalization	0.98 ([0.96–1.00])	0.091	0.98 ([0.96–1])	0.053
Time to Study Drug	0.99 ([0.98–0.99])	0.046	1.00 ([0.99–1])	0.359
Antibiotic Used Before Ciprofloxacin with activity	0.17 ([0.05–0.61])	0.006	0.26 ([0.06–1.17])	0.079

Abbreviations: OR, odds ratio; CI, confidence interval. *^a^* Discrimination (AUC = 0.783) and calibration (Hosmer and Lemeshow χ^2^ = 5.722; *p* = 0.678).

## Data Availability

The data presented in this study are available on request from the corresponding author due to privacy.
